# Acknowledgement of Reviewers, 2021

**DOI:** 10.1200/GO.21.00291

**Published:** 2021-09-20

Peer review is at the core of scientific progress. It is through the careful critique of peer reviewers that papers are scrutinized, evaluated, and improved. A paper that has been peer is determined to be valid and worthy of publication. These peer-reviewed papers are a critical component advancing our existing understanding of science.

As such, scientific outlets in general, and *JCO Global Oncology* in particular, owe an immense debt of gratitude to our volunteer peer reviewers. *JCO GO* strives to provide the highest quality peer review, so in recognition of Peer Review Week 2021 and its theme of Identity in Peer Review, we are pleased to recognize your efforts and thank you for your expertise and time by presenting this list of those who have contributed to our success through peer review.

For your hard work in 2020-2021, our heartfelt thank you!

An asterisk indicates reviewers who have reviewed three or more manuscripts in 2020-2021. Two asterisks indicate reviewers who participated in the ASCO Reviewer Mentoring program. Three asterisks indicate reviewers who participated in the ASCO Editorial Fellowship program.

Innocent Abayo

Ahmed Nadeem Abbasi

George Kamil Abdalla

Reem Abdallah

Nafisa Abdel Hafiez

Raafat Abdel-Malek*

Hikmat Abdel-Razeq

John Tabiri Abebrese

Ghassan Abou-Alfa*

Afua Abrahams

Judith Abrams*

Maria Abriata

Nour Abuhadra**

Dafalla Abuidris

Andrés Acevedo-Melo

Maria Isabel Achatz

Aleesha Adatia*

Babatunde Adedokun

Bolanle Adegboyega

Felipe Ades

Salim Adib

Eric Adjei Boakye

Pragati Advani*

Shailesh Advani*

Aimaz Afrough**

Philippe Aftimos

Ajay Aggarwal

David Agom

Elisa Agostinetto*

Pedro Aguiar Junior

Fahad Ahmed*

Rosina Ahmed

Safia Ahmed

Joanne Aitken*

Leonard Ajah

Zainab Akere

Oladimeji Akinboro*

Murat Akova*

Sercan Aksoy

Shahinoor Aktev

Samer Al Hadidi

Asrar Alahmadi

Raafat Alameddine*

Enrique Alanya Rodríguez

Jacqueline Alcalde-Castro

Thierry Alcindor*

Marwan Al-Hajeili*

Noora Al-Hammadi

Nasir Ali

Mazin Aljadiry

Eman Alkhalawi

Gustavo Almeida*

Teresa Almeida Santos

Fouad Alnajjar

Ahmed Alshewered

Amin Altijani

Christian Alvarez Privado

Shaymaa AlWaheidi

Nada Alwan*

Richard Ambinder

Yavuz Anacak

Chidinma Anakwenze Akinfenwa

Benjamin Anderson*

Kate Anderson

Nicolas Andre

Ritu Aneja

Paola Angelini

Toyin Aniagwu

Rifat Ara*

Sanchia Aranda*

Olujide Arije

Avan Armaghani

Simeon Aruah

Banu Arun

Francis Asamoah

Daniel Zemenfes Ashebir

Arwa Ashoor

Patricia Ashton-Prolla*

Chite Asirwa*

Nissa Askins

Bassel Atassi

Avani Athauda

Aaron Atkinson

Kirsten Austad*

Mohamed Daffalla Awadalla

Shivanshu Awasthi*

Olutosin Awolude*

Burça Aydın*

Olubukola Ayodele*

Govind Babu*

Ludwing Bacon Fonseca

Warren Bacorro

Ahmed Badawy

Bhausaheb Bagal

Saphalta Baghmar

İnci Yaman Bajin

Marieke Bak*

Ellen Baker

Adithya Balasubramanian

Satheesan Balasubramanian

Rosemary Balleine

Hideaki Bando

Pedro Barata*

Sanaa Bardaweel

İbrahim Barışta

Jill Barnholtz-Sloan

Joaquin Barnoya

Ronald Barr

Regina Barragan-Carrillo

Carmelia Barreto

Ute Bartels

Michael Barton

Atul Batra

Nicolò Battisti

Beth Beadle

Brendan Bebington

Torben Becker

Ann Marie Beddoe*

Mohamed Amine Bekadja

Asim Belgaumi*

Martine Bellanger

Javier David Benitez Fuentes**

Sara Benitez Majano*

Charles Bennett

Jessica Bensenhaver

Safedin Beqaj

Anouk Berger

Paulo Bergerot*

Alejandro Berlin*

Scott Berry*

Pascal Bessong

Pierre Bey

Afsan Bhadelia*

Nickhill Bhakta*

Rohini Bhatia

Ami Bhatt

Gouri Shankar Bhattacharyya*

Shristi Bhattarai

Sita Bhella

Ramesh Bilimagga

Ahitagni Biswas

Barri Blauvelt*

Jean-Yves Blay

Prunella Blinman

Amy Body

Oliver Bogler

Renata Bonadio*

Anita Bonthuys

Massimo Bonucci

Christopher Booth*

Margaret Borok Williams

Maarten Bosland

Eric Bouffet*

Fouad Boulos

Maria Bourlon*

Marouen Braham*

Amanda Brahim

Hasan Brau-Figueroa

Paul Brennan

Ari Brooks*

Ewan Brown

Geoffrey Buckle

Henriette Burger*

Mauricio Burotto

Sahai Burrowes*

Sita Bushan**

Anita Bustam

Anna Cabanes*

Paula Cabrera-Galeana

Gabriele Calaminus

German Calderillo

Rolando Camacho*

Conceicao Campos**

Fernando Campos

Saul Campos-Gomez

Miguela Caniza*

Anna Cantor*

Andres Cardona Zorrilla

Christine Carrington

Heloisa Carvalho

Jose Casali da Rocha

Carlos Castaneda

Philip Castle

Carlos Castro

Eduardo Cazap*

Bruce Chabner

Pavani Chalasani

Robert Chamberlain

Alexandre Chan

Guillermo Chantada*

Yanin Chavarri-Guerra*

Lilian Chen

Yingxi Chen

Zhi Cheng

William Cherniak

John Whay Kuang Chia

Alberto Chiappori

Runcie Chidebe*

Fredrick Chite*

Sung-hee Choi*

Toni Choueiri

Eric Chow

Melvin Chua

YuJo Chua

Linus Chuang*

Jeremy Chuang**

Askar Chukmaitov

Harrison Chuwa*

Marcia Ciccone

Carlo Cicero-Oneto

Mishka Cira*

Christina Clarke

Timothy Clay

James Cleary*

Gary Clifford

Graham Colditz

C. Norman Coleman*

Brian Collopy

Michelle Connolly

Stephen Connor

Leigh Conroy

Olivia Cook*

Tim Cooksley

Luis Corrales

Ainhoa Costas-Chavarri

Joanna Coutts

Tracy Crane

Fiona Crawford-Williams

Jennifer Croke*

Mel Valerie Cruz-Ordinario

Heather Cubie

Bernard Cummings*

Alberto Jacobo Cunquero Tomás*

Giuseppe Curigliano

Sanju Cyriac*

Elif Dağlı

Begoña Díaz de la Noval

Neelakanta Dadi

Sameera Dalvie

Anuja Damani

Christopher Dandoy

Harry S. Darling*

Niloy Datta

Amy Davies

Elizabeth Davies

Thomas Davis

Shaheenah Dawood

Daphne Day*

Henry Ddungu

Tiago de Castria

Gilberto de Castro Junior*

Roselle de Guzman

Odin de la Mora

Paul de Souza

Rejane de Souza Reis

Alan DeCherney

Clare Delaney

Lucia Delgado

Kenny Jun Demegillo

Avram Denburg

Lynette Denny

Suryanarayana Deo

Nebiyu Dereje

Amrita Desai*

Aakash Desai

Sampadas Dessai

Baskaran Dhanapal

Haryana Dhillon*

Michelle Dickson

Joanna Didkowska

Helen Dimaras

Omer Dizdar

Nazli Dizman*

Don Dizon

Juan Donaire

Deborah Doroshow

Julia Downing*

Pierre-Antoine Dugue

Narjust Duma*

Nicla La Verde

Elangovan Vidhubala

Arielle Eagan*

Jenny Edge

Muhammad Muzzammil Edhi

Kelechi Eguzo*

David Einstein

Mark Einstein

Uwemedimbuk Ekanem*

Nagi El Saghir*

Moawia Elhassan*

Fadia Elias

Brenda Elias

Elena Elimova

Yannick Eller

Ahmed Elzawawy*

Jesper Grau Eriksen*

Sultan Eser*

Andrea Espejo

Manuel Espinoza-Gutarra

Josee-Lyne Ethier

Mohammed Ezzi

Temidayo Fadelu*

Babatunde Fadipe

Yufei Fan*

Muhammad Mohsin Fareed

Fadi Farhat

Omolara Fatiregun

Pedro Fernandez

Andrew Field*

Brian Fink

Megan Fitzpatrick*

Kenneth Fleming*

Vaia Florou

Lisa Force

Andres Forero

Laura Forrest

Tamer Fouad*

Jane Fox*

Lewis Foxhall

Marie Belle Francia*

Lindsay Frazier*

Sophia Frentzas

Paola Friedrich

Anthony Fyles

Murat Gültekin

James Gajewski

Oscar Galindo Vazquez

Trivadi Ganesan*

Shridar Ganesan

Apar Kishor Ganti

Marina Garassino

Gabriel Garcia

Arun Garg*

Rakesh Garg

Benjamin Garmezy**

Gail Garvey*

Gemma Gatta

Cindy Gauvreau

Edward Gelmann

Sophia George

Sarah Gerdts

Arunangshu Ghoshal*^,^**

Markus Gifoni

Ophira Ginsburg*

Fabio Girardi

Sushil Giri

Angela Giselvania

Meredith Giuliani*

Leslie Given

Rachel Glicksman

Mahesh Goel*

Daniel Goldstein

Susanne Gollin

Mehra Golshan

Rodrigo Goncalves

Soehartati Gondhowiardjo

Jaime Gonzalez

Annekathryn Goodman*

Satsh Gopal*

Sameer Gopalani

William Gradishar

Alois Gratwohl

Marcia Graudenz

Maja Green

Dan Green Renner

Kalinda Griffiths

Rafael Grochot

Thomas Gross

Axel Grothey

Rajesh Grover*

Surbhi Grover*

Bahar Guciz Dogan

Seema Gulia

Derya Gültekin Karakaş

Vishesh Gumdal*

Sumit Gupta

Jason Gurney*

Grace Gwini

Bishal Gyawali*

David Gyorki

Benjamin Haaland

Mahlet Kifle Habtemariam

Neville Hacker

Sabrine Haddad

Asim Hafiz

Ezra Hahn

Khalid Halahleh*

Evan Hall

Daniel Haller

Balazs Halmos

Nazik Hammad*

Kathy Han

Rachel Hanisch

Timothy Hanna*

Jordan Hansford

Prashanth Hari Dass

Mhamed Harif*

Stephen Hawes

Mutlu Hayran*

Volkan Hazar

Valerie Heong

Alexander Heriot

Peter Hesseling

Magda Heunis

Ekkehard Hewer

Gwo-Yaw Ho*

Şefik Hoşal

Jeffrey Hoch

Raymond Hohl

Carlo Hojilla

Susan Horton

Ali Hosni

Marilyn Huang

Megan Huchko*

Winston Huh

Stephen Hunger

Dezheng Huo

Muhammad Husnain

Nzechukwu Ikeri

Chukwuemeka Ikpeazu

André Ilbawi*

Omoruyi Irabor

Mary Isaacson

Muhammad Islam*

Sharif Ismail

Marcin Jacek Jabłoński

Robert Jach

J.C. Kennetth Jacinto

Christopher Jackson*

Aasems Jacob

Geraldine Jacobson

Philipp Jaehn

Maheswari Jaganathan

Ishmael Jaiyesimi

Murali Janakiram

Abdul-Rahman Jazieh

Sima Jeha

Ahmedin Jemal

Michael Jewett*

Ruinuo Jia

Sonali Johnson

Robin Jones

Eric Joo

Deepa Joseph*

Himanshu Joshi

Josephine Kahiu

Dilyara Kaidarova

Aruna Kamimeni

Ravindran Kanesvaran

Yada Kanjanapan

Alfred Karagu

Christos Karapetis

Deme Karikios*

Ahmed Kaseb

Shinichiro Kashiwagi

Babita Kataria

Violet Kayamba*

Jamal Khader*

Uqba Khan*

Shah Khan

Saadiya Khan

Nehal Khanna

Soumen Khatua

Bumyang Kim

Dennis Kim

Peter Kingham*

Nukhet Kirag

Sue Kirsa

Caprice Knapp

Miriam Knoll

Kaladada Korubo*

Eric Krakauer

Robert Kridel*

Rahul Krishnatry

Rajesh KS

Nicole Kuderer*

Jagadish Kudkuli

Vishal Kukreti

Amit Kulkarni

Shaji Kumar*

Rajat Kumar

Abhishek Kumar

Purna Kurkure

Nilgün Kurucu

John Kuruvilla*

Tezer Kutluk*

Edmond Kwan

Bonnie Labdi

Stefanos Labropoulos

Bahar Laderian

Rupa Lakhanpal

Arjun Lakshman

Issam Lalya

Catherine Lam

Ka-On Lam

Luciana Landeiro

Arjun Law*^,^**

Jason Law

Rita Lawlor*

Clara Lee

Leslie Lehmann

Manidhar Reddy Lekkala

Angeline Letendre

Eric Leung

Chi Kong Li

Wenjiao Li

Qingge Li

Ditte Lind

Elizabeth Liow*

Jocelyn Lippey*

Jeffrey H. Lipton*

T. Andrew Lister*

Karolina Lisy

Annemeri Livinalli

Shahin Lockman

Patrick Loehrer

Benjamin Lok

Dorothy Lombe*^,^**

Adhemar Longatto

Florian Lordick

Jonathan Loree

Susan Love

Pak Ling Lui

Caroline Lum

Sandra Luna-Fineman

Maryam Lustberg

Lisa Madlensky

Anazoeze Madu

Vijay Mahobia

Priyamvada Maitre

Carmine Malfitano

Prabhat Malik

Monica Malik

Mohandas Mallath

Adrianne Mallen

Indranil Mallick*

Alexis Manirakiza

Max Mano

Pritchard Mark

Ben Markman

Hugo Martijn

Moses Masika

Deborah Maskens

Maura Massimino

Charbel Matar*

Aju Mathew*

Gustavo Matos**

Luiz Alberto Mattos Jr.

Sam Mbulaiteye*

Kimberly McBride

Nicole McCarthy*

Amelia McCartney*

Bradley McGregor

Itrat Mehdi*

Parth Mehta

Solomon Tessema Memirie

Manoj Menon

Sonia Menon

Sofia Merajver

Emily Merkel

Les Mery

Monika Metzger*

Ralph Meyer

Rafael Meza

Lauta Mezquita

Joseph Mikhael*

Vivienne Milch*

Brittany Miller

Dan Milner*

Michael Milosevic*

Lori Minasian

Mark Minden

Adalberto Miranda-Filho

Kamal Mohamed

Raj Mohan

Michelle Mollica

Cecilia Monge Bonilla

Mariana Monteiro*

Lesley Moody*

Fabio Moraes*

Dion Morton

Kalayu Mruts

Esther Muinga

Deborah Mukherji

Shiva Kumar Mukkamalla

Pablo Munoz Schuffenegger

Gad Murenzi

Tracy Murphy*

Esam Murshid

Shilpa Murthy

Sreenivasa Murthy

Melinda Mushonga

Miriam Mutebi*

Alex Mutombo Baleka*

Catherine Mwaba*

Martin Mynarek

Fumio Nagashima

Mahendra Naidoo

Ntaraj Naidu*

Akira Nakagawara

Elizabeth Namukwaya

Govind Nandakumar

Steven Narod

Gaurav Narula*

Yasodha Natkunam

Rudolph Navari

Kingsley Ndoh

Ann Nelson

Prakash Neupane

Joana Neves

Sweet Ping Ng

Twalib Ngoma*

Mike Nguyen

Tu Nguyen-Dumont*

Wilfred Ngwa*

Claire Nightingale*

Amanda Nogueira*

Fabien Ntaganda

Peter Ntiamoah

Albert Nyamhunga

Folakemi Odedina*

Babatunde Odugbemi

Olufemi Ogunbiyi

Samson Okello

Olufunmilayo Olopade*

Ian Olver

Tanvier Omar

Daniel O'Neil

Tonia Onyeka

Stephen Opat

Jessica Opie

Maria Orozco

Ana Oton

Sai-Hong Ou

Sunday Oyerinde

Vahit Ozmen

Alev Ozon

Lydia Pace*

Matthew Painschab

Sangita Pal

Shani Paluch-Shimon

Rajeev Parameswaran

Wungki Park

Catriona Parker

Robert Parker

Parth Mehta

Moosa Patel

Shivani Patel

Sushmita Pathy

Catherine Patte

Atanu Paul

Astrid Pavlovsky

Nir Peled*

Cesar Perez

Edith Perez

Shahana Pervin*

Anh Pham*

Philip Philip

Luca Pierelli

Leeya Pinder

Marion Pineros

Chelsea Pinnix*

Amanda Piper

Bishesh Poudyal*

Richard Powell

Mandi Pratt-Chapman

Hans Prenen*

Anca Prica

Kathy Pritchard-Jones

Douglas Puricelli Perin

Ibrahim Qaddoumi*

Bilal Mazhar Qureshi

Cristina Rabadan-Diehl

Derek Raghavan*

Akshara Raghavendra**

Ajay Raghunath*

Khadija Rahman

Bhavana Rai

Kanti Rai

Shweta Rai

Shalini Rajaram

Anant Ramaswamy

Patrician Ramirez-Noguera

Sushmita Rath

Vivek Rathi*

Nirmal Raut

Praful Ravi

Evangelia Razis

Timothy Rebbeck

Tony Reiman

Bharat Rekhi

Jordi Remon

Lorna Renner*

Raul Ribeiro

Eli Ristevski

Chad Ritch

Monica Rivera Franco

David Roberge

Mark Robson

Danielle Rodin*

Gary Rodin*

Juliana Rodriguez

Laura Rodriguez

Carlos Rodriguez-Galindo*

Lindsey Roeker

Paul Rogers

Felipe Roitberg*

Anya Romanoff

Daniela Rosa

Davide Rossi

Sevket Ruacan

James Rubenstein

Paul Ruff

Mark Rutherford

Piotr Rutkowski

Danielle Benedict Sacdalan

Dennis Sacdalan

Adrian Sacher

Roya Sadeghi

Muhammad Saghir

Arzu Saglam

Solmaz Sahebjam

Arjun Sahgal

Hector Said

Rakiya Saidu

Mahmoud Salama

Ricardo Sales dos Santos

Coşkun Salman

Suraj Samtani*

Flor Sanchez

Marceli Santos

Taroh Satoh

Hay Mar Saw

Shahin Sayed*

Richard Schilsky

Matthew Schlumbrecht

Rafael Schmerling

Berndt Schmit

Hans-Joachim Schmoll

Kristin Schroeder

Ramy Sedhom

Kartik Sehgal

Vinodh Kumar Selvaraj**

Meltem Sengelen*

Delaram Shakoor

Jaime Shalkow*

Abhishek Shankar

Daya Nand Sharma

Surendra Shastri*

Yuankai Shi

Ramila Shilpakar*

Kohei Shitara

Lawrence Shulman*

David Shultz

Iman Sidhom

Monica S. Sierra

Alben Sigamani

Hannah Simonds*

Simron Singh

Shalini Singh

Bhawna Sirohi*

George Sledge

Jeremy Slone*

Robert Smith

Angela Solano*

Montserrat Soler

Benjamin Solomon

Takayuki Sone

Enrique Soto-Perez-de-Celis*

Melissa Southey*

Anthonia Sowunmi

Dingle Spence

Priyanka Srivastava

Susannah Stanway*

Daniela Stefan*

Simona Stolnicu

Daniel Stover

Simone Strasser

Richard Sullivan*

Iyad Sultan

Rajitha Sunkara

Daniel Surridge

Nao Suzuki

Aaron Sverdlov

Vikas Talreja

Gevorg Tamamyan

Pui San Tan

Nidale Tarek

Marios Konstantinos Tasoulis*

Laure-Anne Teuwen

Michael Thompson

Han Chong Toh

Mazhar Tokgözoğlu

Tamiwe Tomoka

Lindsey Torre

Thuan Tran

Huong Tran

Avraham Travers

Edward Trimble*

Terry Trinh

Derek Tsang

Vivien Tsu*

Siddharth Turkar**

Duygu Uçkan

Adrian Udrea

Thomas Uldrick

Jayant Vaidya*

Claire Vajdic

Omaira Valencia

Nandini Vallath

Jacob Van Dyk*

Ronwyn van Eeden

Linda Van Le

Verna Vanderpuye*

Ali Varan

Cherian Varghese

Jule Vasquez

Vinicius Vazquez

Surendran Veeraiah

Christopher Venner

Shama Virani

Katherine Virgo

Auro Viswabandya

Steven Vogl*

Patricia Volkow

Daniel Vorobiof*

Gordan Vujanic

Horia Vulpe*

Tabassum Wadasadawala*

Sara Wahlroos*

John Waldron

Grace Walpole

Padraig Warde*

Jeremy Warner

Kate Webber*

Jeffrey Weitzel

William William

Brooke Wilson*

Paul Wissel*

Rebecca Wong*

Teresa Woodruff

Ferah Yildiz

Emad Yahaghi

Bilgehan Yalcin*

Suayib Yalcin*

Jennifer Yeh

Kheng Yeoh

Desmond Yip*

Sook Yee Yoon

Hyun Jo Youn

Joanna Young*

Annie Young

Graeme Young

Sema Yurduşen

Muhammed Yusuf

Wafik Zaky

Roberto Zanetti*

Farhad Zangeneh

Ines Zemni

Nik Zeps*

Stenio Zequi

Alona Zer

Hongcheng Zhu

Camilla Zimmermann

Eduardo Zubizarreta*

Jo Anne Zujewski*

Mauro Zukin

